# Association between dopamine receptor D2 *Taq* IA gene polymorphism (rs1800497) and personality traits

**DOI:** 10.1177/20503121241241922

**Published:** 2024-05-14

**Authors:** Holiness S.A Olasore, Tolulope A. Oyedeji, Joseph O. Faleti, Omobola I. Ogundele, Anthony A. Olashore

**Affiliations:** 1 Faculty of Basic Medical Sciences, Department of Biochemistry, College of Medicine of the University of Lagos, Idi Araba, Lagos, Nigeria; 2 Faculty of Medicine, Department of Psychiatry, University of Botswana, Gaborone, Botswana

**Keywords:** Dopamine receptor, gene polymorphism, human personality

## Abstract

**Objective:**

This study aimed to find a potential association between the *DRD2 Taq*1A gene polymorphism (rs1800497 C32806T) and personality traits.

**Methods:**

In all, 249 youths were recruited for this study. The Short-form Revised Eysenck Personality Questionnaire was administered to assess personality traits. The participants were genotyped for the *DRD2 Taq*1A polymorphism using the polymerase chain reaction-restriction fragment length polymorphism method. Statistical analysis was carried out to find a possible association between the genotypes and aspects of personality traits assessed.

**Results:**

The frequencies of the A1 and A2 alleles in our sampled population were 215 (43.2%) and 283 (56.8%), while the frequencies of A1A1, A1A2, and A2A2 were 67 (26.9%), 81 (32.5%), and 101 (40.6%), respectively. The study population was not in Hardy-Weinberg equilibrium (*χ*^2^ = 17.64, *p* < 0.001). The A2 allele was significantly associated with extraversion. Although this allele was also associated with neuroticism, psychoticism, and lie, the association was not significant.

**Conclusion:**

The A2 allele of the *DRD2 Taq*1A polymorphism was found to be more associated with extraversion, as measured by the Short-form Revised Eysenck Personality Questionnaire.

## Introduction

The preservation of normal processes throughout life, behavior, and lifestyle is influenced by personality. Individual differences are based on characteristics connected to behavior, cognition, emotions, and motivation.^1,2^ There are several theoretical models adapted for the complexity of human personality—the biogenetics and the environmental variables that influence personality traits. Examples include the ground-breaking models put out by Eysenck and Eysenck.^3^ The Eysenck personality questionnaire measures Extraversion (*E*), Neuroticism/Anxiety (*N*), and Psychoticism (*P*), as well as incorporating a social desirability scale. Human personalities are multi-gene products. Since behavioral traits are polygenic, the classical genome-wide linkage analysis cannot easily detect the genes responsible for personality traits.^4–6^ Nevertheless, studies on twins illustrated that genetic inheritance explains 73% of extraversion variability.^7^

Eysenck’s theory centers around temperament, which is constituted by innate and genetically determined personality variations.^8^ According to Eysenck, biology is primarily responsible for shaping one’s personality.^8^ He postulated that individuals possess two distinct personality dimensions: extroversion versus introversion and neuroticism versus stability. Extroversion is characterized by sociability, impulsiveness, frivolity, overall activity level, and overt sexuality. Neuroticism, on the other hand, pertains to one’s emotional stability or instability and encompasses mood swings, poor emotional adaptation, feelings of inferiority, lack of social responsibility, lack of perseverance, trust issues versus suspiciousness, social shyness, hypochondria, and lack of relaxed composure. Psychoticism entails traits such as dominance leadership, dominance submission, sensation seeking, and the absence of a superego.^8^

Different personality qualities like extraversion and neuroticism are unswervingly different within and between populations.^9,10^ For example, across populations, variation in extraversion generally accounted for between 3% and 7% of the overall variation, while neuroticism accounted for between 3% and 10%. Asian populations were found to be less extroverted than the European and North American populations, but people in East Asia, Southern Europe, and South America showed higher neuroticism.^9,11^

The Short-form Revised Eysenck Personality Questionnaire (EPQR-S) is a widely recognized personality assessment tool that has gained global popularity. It is a psychometrically sound and theoretically motivated measure that has been extensively employed in various populations and linked to a range of dimensions. This has proved its unambiguous value in the evaluation of personality. The instrument has been validated in many educational, military, health, and forensic areas.^12,13^ Psychometric studies have been conducted on these instruments in many countries, including Nigeria, and different cultures over five continents, and results have confirmed the validity of this instrument.^14–16^

Brain functionality depends on neurotransmission mediated by endogenous substances called neurotransmitters, which control how information is processed and how neurons communicate with one another in the brain.^17^ Biogenic amine neurotransmitters, such as dopamine, norepinephrine, epinephrine, histamine, and serotonin, participate in various types of physiological functions, such as regulating cognitive abilities, mood, etc.^18–20^ According to research, biogenic amines are intriguing possibilities for being key autointoxication mediators that might be implicated in the development of depressive symptoms, anxiety (neuroticism), and perhaps psychosis.^21,22^ Norepinephrine, serotonin, and dopamine have been mostly investigated in relation to antisocial behavior.^23,24^

Numerous studies have linked the dopaminergic system to personality, specifically as it relates to the involvement of certain receptor subtypes, the variable expression of metabolic enzymes, and variations in the function of dopamine transporters.^25^ Hence, dopaminergic genes may be considered logical candidates for characteristics linked to motivating behaviors, such as neuroticism or extraversion. Several correlational and experimental techniques have demonstrated the importance of dopamine for behavioral characteristics of the extraverted and neurotic kind.^26,27^ Some of the most extensively researched polymorphisms in the dopaminergic system concerning behaviors are the dopamine D2 receptor gene polymorphisms.^25,28^

Many variations of the dopamine receptor D2 (*DRD2*) gene have been identified; however, the *Taq*1A polymorphism (rs1800497, C32806T, or Glu713Lys) in the gene’s 3ʹ flanking region has been the focus of most studies.^29^ The first and second exons of the *DRD2* gene are separated by 250 kb intron, and the complete *DRD2* gene is approximately 270 kb long.^30^ The *Taq*1A polymorphism is found inside the *ANKK1* gene, 10,541 bp downstream (C32806T) of the *DRD2* termination codon.^31,32^ This polymorphism alters the density of the dopamine D2 receptor. Compared to non-carriers, A1 allele carriers of the *DRD2 Taq*1A polymorphism had lower striatal D2 receptor densities in the functional areas controlling reward, learning, addiction, and neuroticism.^33^ The *DRD2 Taq*1A polymorphism has been found to significantly influence reward behavior and the brain activity that underpins it.^33^ The strength and longevity of these links are less clear; nonetheless, dopamine gene polymorphisms have been related to variances in personality traits in both clinical and general populations.^27,34^

The dopaminergic system is central to regulating the brain systems that govern cognitive and emotional decision processes underpinning extraversion and neuroticism.^27,35^ Researchers have examined the correlation between *DRD2 Taq*I A polymorphisms and certain personality traits, such as extraversion and neuroticism.^36–38^ However, to our knowledge, no study has examined the relationship between this widely researched and important polymorphism affecting the expression of the dopamine D2 receptor and a combination of aspects of human personality assessed by the popular and well-validated EPQR-S. Therefore, the present study was designed to assess the relationship between DRD2 *Taq*1A polymorphism and personality using the EPQ.

## Materials and methods

### Ethical consideration and subjects

This cross-sectional observational study was carried out following the approval of the Health Research Ethics Committee (HREC) of the College of Medicine of the University of Lagos (with approval number: CMUL/HREC/06/20/745). All participants were required to provide written informed consent. For participants under 18, written parental consent was also obtained. Due to the sensitive nature of human personality, a convenient sampling method was used with 249 participants recruited from the University of Lagos Campus communities (Akoka, Yaba, and Idi-Araba, Surulere) within Lagos State, Nigeria. The total number was made up of 134 young men and 115 young women. Written informed consent was obtained from the participants. Inclusion criteria were willingness to participate in the study and ability to read and understand the questionnaire. Exclusion criteria were self-reported previous diagnosis and/or treatment for any psychiatric illness.

### Personality assessment

The personality trait of participants was assessed using the EPQR-S, a self-report measure of personality based on three dimensions: Extraversion, Neuroticism, Psychoticism, and a Lie scale.^3^ The questionnaire contains 48 randomly arranged questions with YES/NO options designed to test the aforementioned dimensions of personality, and the participants were blinded to the aspect of personality being tested by each question to encourage them to answer the questions as honestly as possible.

A point was awarded to those who answered “Yes” to questions 10, 14, 22, 31, and 39 (for psychoticism); 3, 7, 11, 15, 19, 23, 32, 36, 44, and 48 (for extraversion); 1, 5, 9, 13, 17, 21, 25, 30, 34, 38, 42, and 46 (for neuroticism); and 4, 16, and 45 (for lie) while a point was awarded to those who answered “No” to questions 2, 6, 18, 26, 28, 35, and 43 (for psychoticism); 27 and 41 (for extraversion); and 8, 12, 20, 24, 29, 33, 37, 40, and 47 (for lie).^3^ A maximum score of 12 was obtainable on each scale, while scores from 1 to 4 were categorized as low, 5 to 8 as moderate, and 9 to 12 as high.

### Blood sample collection

A venous blood sample (1 ml) was collected for DNA analysis in ethylenediaminetetraacetic acid bottles by qualified phlebotomists.

### DNA extraction

High molecular weight genomic DNA was prepared from the whole peripheral venous blood samples using a solution-based method using the Blood DNA Preparation Kit (Jena Bioscience, Germany) according to the manufacturer’s protocol. The DNA yield was determined using Nanodrop 1000 (Thermo Scientific, USA). The yield was found to be between 45 and 65 µg/ml of whole blood.

### Genotyping

The participants were genotyped for *DRD2 Taq*1A polymorphism by polymerase chain reaction-restriction fragment length polymorphism (PCR-RFLP) analysis described previously by Olasore et al.^39^ However, with some modifications. The sequences of the primer set used are as follows: forward: 5′-CCG TCG ACG GCT GGC CAA GTT GTC TA-3′ and reverse: 5′-CCG TCG ACC CTT CCT GAG TGT CAT CA-3′. PCR was carried out in a reaction volume of 20 µl, containing 10 µl of 2× master mix with standard buffer (New England Biolabs, USA), 2 µl each of forward and reverse primers, 2 µl of nuclease-free water, and 4 µl of genomic DNA. The PCR cycling profile was initial denaturation at 94°C for 3 min, followed by 35 cycles of denaturation at 94°C for 30 s, annealing at 58°C for 1 min, and extension at 72°C for 2 min, and then a final extension at 72°C for 2 min.

The PCR products (15 µl) were digested for 12 h at 65°C with five units of *Taq1* restriction enzyme (New England Biolabs, USA). The products were electrophoresed on 2.5% gels stained with GelGreen^®^ DNA stain (Biotium, USA). The expected fragments were 310 bp for the whole amplified gene region, which meant the *Taq1* restriction site was absent (A1 allele), while the presence of 180 and 130 bp fragments meant the site was present (A2 allele).

### Statistical analyses

All statistical analyses were carried out using the SPSS version 25 software *(SPSS Inc., Chicago, IL, USA)*. The descriptive statistics, the allele, and genotype distributions were analyzed using frequency estimation according to personality. The chi-square test (*χ*^2^) was applied, and the allele distributions between personalities were also compared. Hardy-Weinberg equilibrium (HWE) was estimated using the chi-square test. The association between the *DRD2* alleles and personality scores was analyzed using binary logistic regression. All *p* < 0.05 were considered statistically significant.

## Results

**Figure 1.**
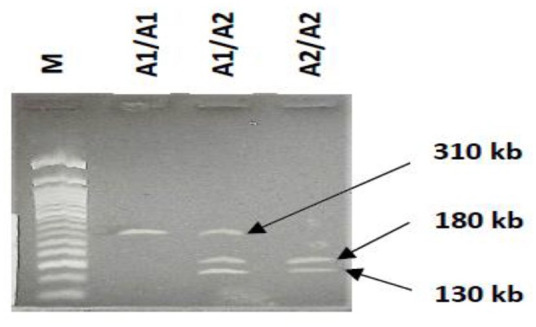
Agarose gel image showing the DNA fragments for different alleles and genotypes. The lane labeled M is for the 50 bp marker. The 310 bp fragment was the whole amplified gene region showing that the *Taq*1 restriction site was absent (A1 allele) while the presence of 180 and 130 bp fragments shows the site was present (A2 allele).

The result, as presented in Table 1, describes the participants in this study. Most participants were within the adolescent age range with a mean age ± SD of 20.70 ± 2.63 years with upper and lower age boundaries of 15 and 31 years. Most participants were male, 134 (53%), while others identified as female. Figure 1 is an agarose gel showing the PCR products’ various fragment sizes representing different alleles and genotypes.

**Table 1.** Descriptive statistics of the sampled population.

Age (mean ± SD) years	20.70 ± 2.63
Age range (years)	15–31
Gender	
Males *n* (%)	134 (53.8)
Females *n* (%)	115 (46.2)

Table 2 shows the distribution of allele and genotype frequencies of the *DRD2* gene based on the *Taq*1A polymorphic site within the sampled population. The A1 and A2 allele frequencies were 215 (43.2%) and 283 (56.8%), respectively, showing that the A2 allele predominated. Also, the frequencies of A1/A1, A1/A2, and A2/A2 were 67 (26.9%), 81 (32.5%), and 101 (40.6%), respectively, showing that A2/A2 genotype was the most frequent of the three. The population was not in HWE with respect to the *Taq*1A site (*χ*^2^ = 17.64, *df* = 1, *p* < 0.001).

**Table 2.** Distributions of alleles and genotypes within the study population.

Allele/Genotype	Frequency (%)		
Allele	A1	A2	
	215 (43.2)	283 (56.8)	
Genotype	A1/A1	A1/A2	A2/A2
	67 (26.9)	81 (32.5)	101 (40.6)
	*χ* ^2^	*df*	*p*
Hardy-Weinberg equilibrium	17.64	1.00	<0.01

Table 3 shows the distribution of the A1 and A2 alleles based on personality scores for all aspects of personality measured. The personality scores were classified as low, moderate, and high. The A2 allele remained the predominant allele on all the personality scales. For all the high score categories, the A2 had higher frequencies, 104 (67.5%), 82 (70.7%), and 26 (59.1%) for extraversion, neuroticism, and lie scales, respectively, compared to the A1 allele. None of the participants fell into the category of a high score on the psychoticism scale. The reverse was the case for the low score categories, where the A1 allele was the predominant allele for all the aspects of personality. Similarly, in the moderate score categories, except for the lie scale where the A1 allele frequency was 107 (44.6%), the A1 allele was more frequent at 106 (50.5%), 12 (75.0%), and 101 (52.6%) for extraversion, psychoticism, and neuroticism scales, respectively, compared to the A2 allele.

**Table 3.** Distribution of *DRD2* alleles according to scores on personality scales.

Allele	Frequency ***n*** (%)			
	Personality scale	Low score	Moderate score	High score
	Extraversion			
A1		59 (44.0)	106 (50.5)	50 (32.5)
A2		75 (56.0)	104 (49.5)	104 (67.5)
	Psychoticism			
A1		203 (42.1)	12 (75.0)	Nil
A2		279 (57.9)	4 (25.0)	Nil
	Neuroticism			
A1		80 (42.1)	101 (52.6)	34 (29.3)
A2		110 (57.9)	91 (47.4)	82 (70.7)
	Lie			
A1	c	90 (42.1)	107 (44.6)	18 (40.9)
A2		124 (57.9)	133 (55.4)	26 (59.1)

The distribution of the genotype frequencies is presented in Table 4. The homozygous A2 allele was the most frequent in the low score category on all the personality scales—27 (40.3%), 101 (41.9%), 39 (41.0%), and 46 (43.0%), respectively. Similarly, apart from the lie scale where the heterozygous genotype predominates, that is, 10 (45.4%), the homozygous A2 was found to be the most frequent genotype in the high score categories—42 (54.5%) and 32 (55.2%) for extraversion and neuroticism, respectively, compared to the other two genotypes. None of the participants was in the high score category on the psychoticism scale.

**Table 4.** Distribution of *DRD2* genotypes according to scores on personality scales.

Genotype	Frequency ***n*** (%)			
	Personality scale	Low score	Moderate score	High score
	Extraversion			
A1/A1		19 (28.4)	33 (31.4)	15 (19.5)
A1/A2		21 (31.3)	40 (38.1)	20 (26.0)
A2/A2		27 (40.3)	32 (30.5)	42 (54.5)
	Psychoticism			
A1/A1		63 (26.1)	4 (50.0)	Nil
A1/A2		77 (32.0)	4 (50.0)	Nil
A2/A2		101 (41.9)	0 (0.0)	Nil
	Neuroticism			
A1/A1		24 (25.3)	35 (36.5)	8 (13.8)
A1/A2		32 (33.7)	31 (32.3)	18 (31.0)
A2/A2		39 (41.0)	30 (31.2)	32 (55.2)
	Lie			
A1/A1		29 (27.1)	34 (28.3)	4 (18.2)
A1/A2		32 (29.9)	39 (32.5)	10 (45.4)
A2/A2		46 (43.0)	47 (39.2)	8 (36.4)

Table 5 presents a binary regression analysis of the relationship between the two alleles and scores on the various aspects of personality. We first classified personality outcome variables into low- and high-score categories for analysis. Only the A2 allele significantly correlated (*p* = 0.047) with extraversion scores (COR = 1.68; 95% CI: 1.01–2.79). Although there was some correlation between the alleles and other aspects of personality measured, these were not all significant. We could not analyze for psychoticism as all participants fell into a single category of low scores; therefore, we did not present this.

**Table 5.** Binary logistic regression analysis showing the association between *DRD2* alleles and personality scores.

**Allele**	**Personality scale**	**SE**	**Wald**	**Sig**	**COR**	**95% CI**	
						Lower	Upper
	Extraversion						
**A2**		0.26	3.93	0.047	1.68	1.01	2.79
	Neuroticism						
**A2**		0.26	1.44	0.230	1.37	0.82	2.28
	Lie						
**A2**		0.29	0.00	0.959	1.02	0.57	1.80

## Discussion

The present study has examined the relationship between *DRD2 Taq*1A gene polymorphism and personality traits among youths in Lagos, Nigeria. The mean age of the participants in this study was within the adolescent age range, which was similar to what was reported in a study by Smillie et al.^36^ The gender distribution in our study is also similar to what they reported in their study. The allelic distribution in this study was in line with what was previously reported for the nonsmoking population in our previous study, comparing the distribution of the *Taq*1A alleles of the *DRD2* between cannabis smokers and nonsmokers.^39^

Another study conducted on the association of cognitive performance and genetic variance of *DRD2 Taq*1A by Tan and Lim.^40^ using a similar population sample was also consistent with the allelic distribution in this study. A meta-analysis study on normal populations in Europe and Asia found that the A1 allele ranged from 6% to 44%, and the higher frequencies for the A2 allele are consistent with our findings.^41^ However, the observed genotype frequencies in this population study were not in HWE, indicating the frequencies were not constant over time. This deviation from the HWE could be due to forces such as ethnicity, population stratification, environment, and natural selection.^42,43^

It has been reported that the *DRD2 Taq*1A alleles influenced personality traits, with the A1 allele being associated with higher extraversion and neuroticism scores.^44^ However, the opposite was the case in our study, with the A2 allele showing more association with higher extraversion and neuroticism scores. According to Baik et al.,^45^ there is a significant positive correlation between dopaminergic receptor availability in the striatum and extraversion. The pattern of our result is attributable at least partly to the fact that the A2 allele is associated with higher striatal D2 receptor availability.^33^ Although extroversion has been associated with the hyperdopaminergic state, D2 receptor density has also been linked to neuroticism. The increased dopaminergic receptor availability and its neurophysiological effects in the striatum suggested increased task-dependent dopaminergic neurotransmission in extroverts.^33,37,46^ It is generally assumed that those who carry the A1 allele experience a reduced sensitivity to reward as a result of a 30%–40% reduction in D2 receptor density in the striatal region compared to the A2 allele, which is well known for a hyperdopaminergic state.^45^

Eysenck suggested that people with neurotic traits are very sensitive to stress and, therefore, tend to experience fear and anxiety.^47^ Ozkaragoz and Noble^38^ reported that children homozygous for the A2 allele from a nonalcoholic home with less stress showed greater scores in the expression of extraversion, although not significant. On the contrary, the A1 allele was associated with higher extraversion among children from alcoholic homes who were more stressed. They hypothesized that a less stressful environment is associated with greater extraversion in children with the major allele (A2) of the *DRD2* gene. An explanation for this is that the dopaminergic system, which is highly affected by stress, plays a key role in an organism’s defensive response toward aversive stimuli, which is dependent on the genetic constitution. They suggested that subjects with the *DRD2* minor (A1) allele may cope with a stressor by increasing their level of activity.^38^ This increase in *DRD2* activity will, in turn, create more reward-mediated traits, as explained earlier, which suggests why other studies found A1 more associated with extraversion and neuroticism. On the other hand, A2 allele carriers are already hyper-dopaminergic and might not need a coping mechanism in a stressful situation, which may be the reason for the increased association of the A2 with extraversion and neuroticism scores in this study. The above confirms genetic factors appeared to intensify extraversion and neuroticism among those exposed to a stressful environment. This suggests that a gene–environment interaction is a factor for higher extraversion and neuroticism scores among carriers of the A2 allele in this study.

Our research indicates that there is no significant correlation between the two alleles and psychoticism scores among our study participants. It is noteworthy that all participants exhibited low scores on the psychoticism scale, which may be attributed to their overall good mental health. However, individuals who carry the A2 allele have shown a tendency toward lower psychoticism scores. It is important to note that high levels of psychoticism are linked to various mental disorders, including manic depression and schizophrenia, but a high score on the psychoticism scale is not necessarily indicative of a psychotic disorder.^47^ Upregulation of the D2 high receptors is a recurring characteristic in animal models of schizophrenia.^48^ This increase in D2 receptors seems to underlie the development of psychosis, and it is associated with dopamine sensitivity.^49^ This particular increase in D2 receptors and dopamine hypersensitivity may result in the failure of antipsychotic therapy.^50^ Changes in D2 receptor function mediated by antipsychotic treatment in a rodent model of schizophrenia or by the administration of amphetamine in schizophrenia patients have recently been observed.^51,52^ While sustained high D2 receptor occupancy is not necessary for the positive effects of antipsychotics,^53,54^ a D2 receptor occupancy of 80% is thought to be necessary.^50^ Schizophrenia patients with extrapyramidal syndromes (EPSs) had higher D2 receptor occupancy (over 80%) than schizophrenia patients with a strong clinical response and no EPSs (i.e., receptor occupancy of 65%–80%).^55^

It has been suggested that the A1 allele is associated with a mutation in the promoter/regulatory gene element that decreases the D2 receptor expression.^56,57^ Although this study did not measure the density of the D2 receptors in individuals carrying the A2 allele, other studies have linked high DA receptor density to people carrying the A2 allele.^58^ Reuter et al.^59^ reported a high DA receptor density for people homologous for the A2 allele (hyperdopaminergic) and low receptor density associated with individuals carrying the A1 allele,^59^ predicting that this may have an impact on the high behavioral approach system.

To the best of our knowledge, studies have not linked the EPQR-S lie scale to the *DRD2 Taq*1A alleles. Nevertheless, studies have shown a correlation between extraversion, neuroticism, and the lie scale.^60^ There has been a significant negative correlation between neuroticism scores and lie scores, suggesting that the lie scale functions as an index of “faking good.”^61,62^ The lie scale was reported to correlate positively with extraversion, although the correlation was weak.^62,63^ Our finding did not confirm this correlation, but it can be inferred that carriers of the A2 allele are more prone to “faking good” than those with the A1 allele. We believe that future research will justify these findings.

However, the findings from this study should be interpreted with caution due to the study’s limitations, such as the sample size. We could not calculate the sample size because everyone, to various extents, possesses the variable we measured, and therefore, the prevalence was 100%, as revealed in our pilot study. The study’s scope is limited due to the absence of participants from diverse geographical locations, age brackets, educational backgrounds, and socioeconomic levels. This limitation may impact the generalizability of the study’s findings and the extent to which they can be applied to other populations. Therefore, future research should aim to include a more diverse sample to enhance the study’s external validity and broaden its applicability.

## Conclusion

In conclusion, we found an association between the *DRD2 Taq*1A gene polymorphism and some aspects of personality. Carriers of the A2 allele were found to exhibit significantly higher scores on the extraversion scale of the EPQR-S. Although carriers of this allele were also found to score higher on neuroticism scales, this was insignificant. There was no association between the *Taq*I A polymorphisms and psychoticism. Personality traits are often influenced by two variables—biogenetics and environment. As observed in this study, the effect of A2 on personality traits could have been influenced by the environment. We recommend a larger sample size to increase the statistical power and a random sampling method in subsequent studies. Future studies should also consider the other sociodemographic variables and other extremes of age in establishing the effect of DRD2 *Taq*1A polymorphism on personality traits in different populations.

## Supplemental Material

sj-docx-1-smo-10.1177_20503121241241922 – Supplemental material for Association between dopamine receptor D2 Taq IA gene polymorphism (rs1800497) and personality traits Supplemental material, sj-docx-1-smo-10.1177_20503121241241922 for Association between dopamine receptor D2 Taq IA gene polymorphism (rs1800497) and personality traits by Holiness S.A Olasore, Tolulope A. Oyedeji, Joseph O. Faleti, Omobola I. Ogundele and Anthony A. Olashore in SAGE Open Medicine
