# Supplementary material for: Association between dopamine receptor D2 Taq IA gene polymorphism (rs1800497) and personality traits
Source: SAGE Open Med. 2024 May 14;12:20503121241241922. doi: 10.1177/20503121241241922 (PMC11095179; doi:10.1177/20503121241241922)
Supplement: sj-docx-1-smo-10.1177_20503121241241922 – Supplemental material for Association between dopamine receptor D2 Taq IA gene polymorphism (rs1800497) and personality traits [file sj-docx-1-smo-10.1177_20503121241241922.docx]

**Participant’s number ___________ Age_________ Sex____________**

**Have you ever been diagnosed or treated for any psychiatric illness in the past?**

**Yes_______ No _______**

**If your answer to the above is Yes, kindly specify the type of illness ___________________________**

**___________________________________________________________________________________**

**PERSONALITY QUESTIONNAIRE**

**(SHORT-FORM REVISED EYSENCK PERSONALITY QUESTIONNAIRE)**

|  | **Extraversion** | | YES | NO |
| --- | --- | --- | --- | --- |
| 3 | Are you a talkative person? | |  |  |
| 7 | Are you rather lively? | |  |  |
| 11 | Do you enjoy meeting new people? | |  |  |
| 15 | Can you usually let yourself go and enjoy yourself at a lively party? | |  |  |
| 19 | Do you usually take the initiative in making new friends? | |  |  |
| 23 | Can you easily get some life into a rather dull party? | |  |  |
| 27 | Do you tend to keep in the background on social occasions? | |  |  |
| 32 | Do you like mixing with people? | |  |  |
| 36 | Do you like plenty of bustle and excitement around you? | |  |  |
| 41 | Are you mostly quiet when you are with other people? | |  |  |
| 44 | Do other people think of you as being very lively? | |  |  |
| 48 | Can you get a party going? | |  |  |
|  | **Neuroticism** | |  |  |
| 1 | Does your mood often go up and down? | |  |  |
| 5 | Do you ever feel ‘just miserable’ for no reason? | |  |  |
| 9 | Are you an irritable person | |  |  |
| 13 | Are your feelings easily hurt? | |  |  |
| 17 | Do you often feel ‘fed-up’? | |  |  |
| 21 | Would you call yourself a nervous person? | |  |  |
| 25 | Are you a worrier? | |  |  |
| 30 | Would you call yourself tense or ‘highly strung’? | |  |  |
| 34 | Do you worry too long after an embarrassing experience? | |  |  |
| 38 | Do you suffer from ‘nerves’? | |  |  |
| 42 | Do you often feel lonely? | |  |  |
| 46 | Are you often troubled about feelings of guilt? | |  |  |
|  | **Lie** | |  |  |
| 4 | If you say you will do something, do you always keep your promise no matter  how inconvenient it might be? | |  |  |
| 8 | Were you ever greedy by helping yourself to more than your share of anything? | |  |  |
| 12 | Have you every blamed someone for doing something you knew was really your fault? | |  |  |
| 16 | Are all your habits good and desirable ones? | |  |  |
| 20 | Have you ever taken anything (even a pin or button) that belonged to someone else? | |  |  |
| 24 | Have you ever broken or lost something belonging to someone else? | |  |  |
| 29 | Have you ever said anything bad or nasty about anyone? | |  |  |
| 33 | As a child were you every cheeky to your parents? | |  |  |
| 37 | Have you ever cheated at a game? | |  |  |
| 40 | Have you ever taken advantage of someone? | |  |  |
| 45 | Do you always practice what you preach? | |  |  |
| 47 | Do you sometimes put off until tomorrow what you ought to do today? | |  |  |
|  | **Psychoticism** | |  |  |
| 6 | | Would being in debt worry you? |  |  |
| 10 | | Would you take drugs which may have strange or dangerous effects? |  |  |
| 14 | | Do you prefer to go your own way rather than act by the rules? |  |  |
| 18 | | Do good manners and cleanliness matter much to you? |  |  |
| 22 | | Do you think marriage is old-fashioned and should be done away with? |  |  |
| 26 | | Do you enjoy co-operating with others? |  |  |
| 28 | | Does it worry you if you know there are mistakes in your work? |  |  |
| 31 | | Do you think people spend too much time safeguarding their future with savings and insurance? |  |  |
| 35 | | Do you try not to be rude to people? |  |  |
| 39 | | Would you like other people to be afraid of you? |  |  |
| 43 | Is it better to follow society’s rules than go your own way? | |  |  |
